# The Presence of Ganglionic Acetylcholine Receptor Antibodies in Sera from Patients with Functional Gastrointestinal Disorders: A Preliminary Study

**DOI:** 10.3390/jpm14050485

**Published:** 2024-04-30

**Authors:** Shunya Nakane, Akihiro Mukaino, Yoshiaki Okumura, Hiroaki Hirosawa, Osamu Higuchi, Hidenori Matsuo, Mosaburo Kainuma, Yuji Nakatsuji

**Affiliations:** 1 Department of Neurology, Faculty of Medicine, University of Toyama, Toyama 930-0194, Japan; 2 Department of Neurology and Clinical Research, National Hospital Organization Nagasaki Kawatana Medical Center, Nagasaki 859-3615, Japanmatsuo.hidenori.wa@mail.hosp.go.jp (H.M.); 3 Department of Neurology, Graduate School of Medical Sciences, Kumamoto University, Kumamoto 860-8556, Japan; 4 Department of Japanese Oriental Medicine, Graduate School of Medicine and Pharmaceutical Sciences, University of Toyama, Toyama 930-0194, Japan; 5 Okumura Forest-All Clinic, Otsu 520-0831, Japan

**Keywords:** functional gastrointestinal disorders, irritable bowel syndrome, functional dyspepsia, anti-ganglionic acetylcholine receptor, autoantibody

## Abstract

Background: Functional gastrointestinal disorders (FGIDs), including functional dyspepsia (FD) and irritable bowel syndrome (IBS), are characterized by chronic and recurrent gastrointestinal symptoms. Clinically, FD and IBS often resemble gastrointestinal dysmotility caused by autoimmune autonomic neuropathy. We examined the seropositive frequency of autoantibodies against ganglionic nicotinic acetylcholine receptors (gnAChRs) in patients presenting with FGIDs. Objective: To elucidate the seropositivity of gnAChR antibodies and the clinical features of seropositive FD and IBS. Materials and Methods: We measured autoantibodies against the gnAChR α3 and β4subunits using luciferase immunoprecipitation systems. Serum samples from patients with any autonomic symptoms were obtained from hospitals in Japan between January 2012 and August 2018 (1787 serum samples of 1381 patients). We selected FD and IBS patients and compared the clinical characteristics and prevalence of autonomic symptoms between those with seropositive and seronegative IBS and FD. Results: Nine IBS and two FD cases (one comorbid case with IBS) were found. We found four patients (36.4%) in whom gnAChR antibodies were positive in these eleven patients. Sicca symptoms were observed in three of four cases (75%) of seropositive FGID compared with zero of seven cases (0%) of seronegative FGID. Conclusions: We found patients with gnAChR antibodies in FD and IBS patients. These data will be valuable for elucidating the pathophysiology of these FGIDs and developing new treatment strategies.

## 1. Introduction

Functional dyspepsia (FD) and irritable bowel syndrome (IBS) are the most common functional gastrointestinal disorders (FGIDs), affecting approximately 20% of the general population [1]. FGIDs are chronic or recurrent diseases in which abnormal bowel movements involving abdominal pain, diarrhea, and constipation, as well as gastric pain and early satiety, persist despite the absence of an organic disease on examination [2]. Similarly, autonomic neuropathy is a condition that presents functional impairment without organic abnormalities. We have previously performed clinical and basic studies of autoimmune autonomic ganglionopathy (AAG), in which autoantibodies against ganglionic nicotinic acetylcholine receptors (gnAChRs) are found in the serum of patients and play a key role in the pathogenesis of the disease [3,4]. Recently, the concept of autoimmune gastrointestinal dysmotility (AGID) has been proposed as a limited form of AAG [5,6]. AGID is becoming a broad concept that includes esophageal achalasia, diffuse esophageal spasm, gastroparesis, and intestinal pseudo-obstruction [7]. Although FD and IBS are clinically similar to the upper and lower gastrointestinal dysmotility in AAG or AGID, the relationship between the pathogenicity and gnAChR antibodies in FGIDs remains unresolved. Hence, we aimed to examine the seropositivity of gnAChR antibodies and the clinical characteristics of seropositive patients with FD and IBS.

## 2. Materials and Methods

### 2.1. Patient Cohort and Study Design

Our institution established the detection system of gnAChR α3 and β4 antibodies by a luciferase immunoprecipitation system (LIPS) assay in 2011, and since 2012, we have responded to requests for assays using serum samples from patients with any autonomic symptoms who visited a hospital in Japan [8,9]. We examined 1787 serum samples of 1381 patients with any autonomic symptoms who visited teaching and general hospitals throughout Japan between January 2012 and August 2018 [8,9]. The serum samples were centrifuged at 3000 rpm for 10 min and were then stored in cryovial tubes at −80 °C within 2 h of collection. The samples were later sent to Nagasaki Kawatana Medical Center or Kumamoto University Hospital.

### 2.2. Luciferase Immunoprecipitation System (LIPS) Assay for Anti-gAChR Abs

In the present study, we detected serum gnAChRα3 and gnAChRβ4 antibodies using the LIPS assay [9]. A National Institutes of Health group previously developed this efficient quantitative approach for the analysis of antibodies against human autoantigens in serum samples from patients [10,11]. We previously established and reported the use of the LIPS to diagnose AAG on the basis of IgGs to both α3 and β4 gnAChR subunits in serum samples from patients [9]. We measured the gnAChR antibodies at the Nagasaki Kawatana Medical Center and Kumamoto University Hospital, as previously described.

To generate luciferase reporters for the human gnAChR subunits, α3 and β4 (named gnAChRα3-GL and gnAChRβ4-GL, respectively) or full-length human gnAChRα3 (P32297; Promega Corporation, Madison, WI, USA) or gnAChRβ4 (P30296; Promega Corporation) were fused to a Gaussian luciferase (GL) mutant (GL^8990^). Human embryonic kidney 293F cells (Life Technologies Corporation, Grand Island, NY, USA) were then transfected with an expression plasmid encoding either gnAChRα3-GL or gnAChRβ4-GL using FuGENE6 (Promega Corporation). The transfected cells were solubilized 2 days later using Tris-based saline containing 1% Triton^TM^ X-100. To detect gnAChRα3 or gnAChRβ4 antibodies, 100 μL of the soluble fraction containing gnAChRα3-GL or gnAChRβ4-GL was incubated with 15 μL of human serum for 1 h at 4 °C. The fraction was then mixed with 15 μL of protein G-Sepharose (GE Healthcare, Little Chalfont, Buckinghamshire, UK) and 600 μL of phosphate-buffered saline with 3% bovine serum albumin and 0.05% Tween^®^-20, and the mixture was incubated for several hours at 4 °C. After centrifugation and two washes with phosphate-buffered saline containing 0.05% Tween^®^-20, the bioluminescence activity of the luciferase reporters in protein G-Sepharose was measured using a BioLux^®^ GL Assay Kit (New England Biolabs, Ipswich, MA, USA) and a Lumat LB 9507 luminometer (Berthold Technologies GmbH & Co. KG, Bad Wildbad, Germany). The luminometer output was measured in relative luminescence units. Using anti-gnAChRα3 and anti-gnAChRβ4 antibody data from 73 healthy controls, cut-off values were calculated as the means + three standard deviations from the mean, as in a previous study. To evaluate the diagnostic accuracy of this LIPS assay, we verified the cut-off values for all data collected in previous studies [12,13]. Cut-off values for the sensitivity and specificity, as well as receiver operating characteristic curves, were obtained. According to these curves, we confirmed the most accurate cut-off values and calculated their sensitivity, specificity, and positive and negative predictive values. The area under the curve was 0.849 (95% confidence interval [CI]: 0.786–0.911) for the LIPS assay of the anti-gnAChRα3 antibody. For an anti-gnAChRα3 antibody cut-off value of 1.0, the sensitivity and specificity values were 46.9% (95% CI: 33.7%–60.6%) and 99.2% (95% CI: 94.8%–100.0%), respectively, whereas the positive and negative predictive values were 95.8% and 81.8%, respectively. The area under the curve was 0.72 (95% CI: 0.632–0.807) for the LIPS assay of the anti-gnAChRβ4 antibody. For an anti-gnAChRβ4 antibody cut-off value of 1.0, the sensitivity and specificity values were 14.3% (95% CI: 6.9%–27.1%) and 100.0% (95% CI: 96.2%–100.0%), respectively, whereas the positive and negative predictive values were 100.0% and 74.4%, respectively.

The antibody levels were expressed as an antibody index (AI), which was calculated as follows: AI = (measured value in the serum sample [in relative luminescence units])/(cut-off value [in relative luminescence units]). The normal AI value, established based on data from healthy individuals, is <1.0. The diagnosis of FD and IBS was confirmed using the Rome IV diagnostic guidelines [14,15]. Clinical diagnoses were performed in each hospital. We compared the clinical data and the prevalence of autonomic symptoms between seropositive and seronegative FD and IBS patients.

### 2.3. Ethical Approval

All patients provided written informed consent for the storage and use of their serum and clinical information for research purposes. The Human Ethics Committees at the Nagasaki Kawatana Medical Center and Kumamoto University Hospital (Japan) approved this study (approval numbers 2011-21 and 1281, respectively).

### 2.4. Clinical Assessment

Specific questionnaires and consent forms were sent to the physicians who referred us to patients, and the data were sorted and analyzed. The questionnaire consisted of six categories with the following entries: (1) age, sex, clinical diagnosis, age at onset of disease, antecedent infection, and mode of symptom onset; (2) autonomic manifestations described below; (3) extra-autonomic manifestations (sensory disturbance, motor symptoms, deep tendon reflexes, gait, and other neurological findings); (4) comorbid diseases (endocrine disorders, tumors, and autoimmune diseases); (5) autonomic testing; (6) other laboratory findings. Regarding the mode of symptom onset, acute onset and subacute onset were defined as reaching peak autonomic symptoms within 3 months. Chronic onset was defined as reaching peak autonomic symptoms after 3 months.

We determined the presence or absence of the following functions controlled by the autonomic nervous system, as reported in our previous study: syncope or orthostatic hypotension and orthostatic intolerance; arrhythmia; pupillary dysfunction; sicca complex; coughing episodes; skin dryness or hypohidrosis/anhidrosis indicating heat intolerance; upper gastrointestinal system problems; diarrhea or constipation indicating dysfunction of the lower gastrointestinal system; dysuria or urinary retention needing catheterization for bladder dysfunction; and sexual dysfunction [8]. We selected patients who were diagnosed with FD or IBS from the patient cohort and divided them into gnAChR antibody-positive and gnAChR antibody-negative groups for a comparative analysis of their clinical features.

### 2.5. COMPASS

Patients with FD or IBS enrolled after April 2014 completed a self-administered questionnaire. COMPASS is a shortened version of the Composite Autonomic Symptom Score and was designed to quantitatively assess autonomic symptoms [16]. It has six subscale weighted scores in the following domains: orthostatic intolerance (four items; range, 0–40), vasomotor (three items; range, 0–5), secretomotor (four items; range, 0–15), gastrointestinal (12 items; range, 0–25), bladder (three items; range, 0–10), and pupillomotor (five items; range, 0–5). The COMPASS assessment is weighted according to published scoring methods to yield a total score of 0–100, with a score of 100 representing the highest, most severe degree of autonomic symptom burden. The mean ± standard deviation score in healthy control subjects for this questionnaire was reported as 9.67 ± 8.1 [17]. In the present study, 11 subjects completed the Japanese language version of the questionnaire within 15 min. We excluded questions related to the vasomotor and pupillomotor domains because it is occasionally difficult for Japanese people to judge color changes of the skin on an individual basis, and it is not the custom for middle-aged and older persons to wear sunglasses or tinted glasses in Japan. The total scores were calculated by the summation of the individual item scores, with a possible maximum score of 90 [8,18].

## 3. Results

In this study, we identified two patients with FD and nine patients with IBS in 1381 patients. One of the former patients also had IBS. Among those 11 patients, 4 patients had gnAChR antibodies. Of the four patients with gnAChR antibody-positive FGID, three had IBS only, and one patient had coexistent FD and IBS. Single seropositivity for gnAChR α3 antibodies was observed in two patients, while single seropositivity for gnAChR β4 antibodies was not observed. Two of the four samples were positive for both gnAChR α3 and β4 antibodies. One of these double-positive patients was particularly refractory to the clinical manifestations of IBS.

The clinical features of the four patients in the gnAChR antibody-positive group and the seven patients in the antibody-negative group were compared, as shown in Table 1. Many of the items that were compared, including the usual epidemiological items and each autonomic symptom, as well as the COMPASS total and domain-specific scores (Table 2), showed no significant differences. Only sicca symptoms were significantly more frequent in the antibody-positive group (75% vs. 0%, *p* = 0.042).

### Illustrative Cases

Patient 1. An 85-year-old man had been affected by IBS for at least 5 years and experienced abdominal pain, constipation, and abdominal bloating soon after eating. The patient did not have orthostatic hypotension/intolerance and had objective findings of dry mouth and upper and lower gastrointestinal dysmotility. The COMPASS also reflected these clinical findings. The patient had only autoantibodies against gnAChRα3, and the serum levels of the gnAChR autoantibodies were 1.017 antibody index (A.I.) (α3) and 0.641 A.I. (β4).

Patient 2. A 51-year-old woman had been affected by FD and IBS for at least 5 years and had nausea, vomiting, and diarrhea. She visited many hospitals and received multiple antiemetic prescriptions, but the nausea remained. She took anti-diarrheal medicines year-round for chronic diarrhea that was triggered by more intense diarrhea that occurred when she consumed high-fat meals. She also had severe left back pain after eating, which led her previous healthcare providers to suspect chronic pancreatitis, and she underwent endoscopic ultrasonography and other tests that did not reveal any abnormalities. An overactive bladder had also been diagnosed, and incontinence was a rare occurrence because of an inability to hold back urine. The patient had autoantibodies for both gnAChRα3 and β4, and her serum levels of the gnAChR autoantibodies were 2.218 A.I. (α3) and 1.135 A.I. (β4).

Patient 3. A 75-year-old woman had been affected by IBS for several years and experienced constipation, appetite loss, nausea, and vomiting. She complained of numbness in both lower extremities and pain in her buttocks in addition to the symptoms attributed to gastrointestinal dysmotility. Seeking further examination and treatment for these symptoms, she consulted a neurologist as well as a local orthopedic surgeon and a pain clinic and was eventually diagnosed with fibromyalgia on the basis of these symptoms. The patient consistently complained of bloating, which was exacerbated by drugs used to treat fibromyalgia, which caused nausea, making it difficult for us to treat both the gastrointestinal dysmotility and fibromyalgia. The patient had autoantibodies for both gnAChRα3 and β4, and her serum levels of the gnAChR autoantibodies were 1.444 A.I. (α3) and 1.078 A.I. (β4).

Patient 4. A 75-year-old woman had been affected by IBS for at least 2 years and experienced abdominal pain, diarrhea, constipation, and abdominal bloating soon after eating. The patient had abdominal pain and discomfort and constipation for several days, followed by diarrhea for several days; therefore, constipation and diarrhea appeared alternately. During the constipation, her abdomen was tense and painful. We prescribed various medications, including Chinese herbal medicines, for the constipation and diarrhea. The patient sometimes had sudden diarrhea when she was away from home; hence, she was afraid to go out. The patient had only autoantibodies against gnAChRα3, and her serum levels for gnAChR autoantibodies were 1.214 A.I. (α3) and 0.175 A.I. (β4).

## 4. Discussion

This preliminary study yielded two findings. First, some of the patients diagnosed with FGID had gnAChR antibodies, and second, the clinical symptom sicca complex was frequently observed in gnAChR antibody-positive FGID patients. These results raise the question of whether gnAChR antibody positivity is involved in the pathogenesis of FGID patients or is coincidental. However, further issues remain to be explored in future research. Although 4 of the 11 patients with FGID in the current study were positive for gnAChR antibodies, it was difficult to determine whether they showed a consistent trend based on age or sex. It was also difficult to determine whether higher levels of gnAChR antibodies or autoantibody positivity regarding both subunits were associated with the severity of abdominal symptoms.

Gastrointestinal symptoms are common and highly prevalent. However, not all patients presenting with gastrointestinal symptoms have a specific organic etiology. Some cases involve FGIDs, such as FD or IBS, in which patients with these conditions suffer from chronic and fluctuating symptoms. The pathogenesis of FGIDs has been described as a gut–brain interaction disorder, which has been studied regarding a variety of aspects, including movement disorders, visceral hypersensitivity, altered mucosal and immune function, altered gut microbiota, and altered central nervous system processing [2,15]. Because the results of the present study suggest the presence of some antibodies in FGID patients, we must consider the possibility that the pathology is mediated by dysimmune status. Atopic and autoimmune diseases, independent of psychological distress, were reported to be risk factors for FGIDs, with psoriasis and rheumatoid arthritis in particular being independent risk factors for IBS [19]. Rheumatoid arthritis was also significantly associated with IBS in another survey of 850 pairs of Swedish twins aged 18–85 years [20]. The involvement of neuro-immune interactions in FGID has been discussed previously [21,22,23,24], and various basic studies have been conducted on the involvement of specific autoantibodies, showing positive and negative results regarding their presence [25,26,27,28,29]. Further detailed clinical and basic studies are needed to determine whether the gnAChR antibodies found in this preliminary study are pathogenic autoantibodies in FGID patients.

Here, we present a relatively new disease concept, autoimmune gastrointestinal dysmotility, a limited form of AAG, which is an autonomic disorder in which gastrointestinal motility disorders are in the foreground of the clinical presentation [5,6,7]. Previous reports have indicated that gastrointestinal dysmotility, such as constipation, diarrhea, alternating constipation–diarrhea, and ileus, as well as orthostatic hypotension and orthostatic intolerance, occur frequently in AAG patients who test positive for gnAChR antibodies, a known pathogenic autoantibody in AAG [7,18]. Although its name implies a localized condition, this disease can present with varying degrees of symptoms from other autonomic domains [7,18]. Another clinical feature of AAG is the presentation of extra-autonomic manifestations, including a tendency to coexist with autoimmune diseases such as autoimmune rheumatic diseases and autoimmune thyroid diseases [8]. Interestingly, complications of gastrointestinal dysmotility in autoimmune rheumatic diseases, such as Sjögren’s syndrome, have been frequently presented in practice [7,30]. Because the present study showed a significantly increased frequency of the sicca complex in patients with gnAChR antibody-positive FGID, we must consider the possibility that these cases were actually autoimmune gastrointestinal dysmotility, a limited form of AAG.

In the peripheral autonomic ganglia, nAChRs (equivalent to gnAChRs) are expressed by neurons in sympathetic, parasympathetic, and enteric ganglia. Patients with AAG often harbor autoantibodies against gAChRs, which may disrupt synaptic transmission in autonomic ganglia and lead to autonomic failure [31,32]. However, the pathogenicity of the gnAChR antibodies, i.e., how the autoantibodies cause autonomic dysfunction, is not entirely clear. More recently, autoantibodies targeting neurotransmitter receptors and related proteins have emerged as an often severe but treatable cause of neurological disease [33]. Autoantibodies against nAChRs in autonomic ganglia should be considered similar to autoantibodies against neurotransmitter receptors when discussing pathogenesis, although AAG is also often present in refractory cases. Previous animal model studies of AAG and an in vitro study using the nAChRα3 subunit expressed in human embryonic kidney cells have shown that autoantibodies for the nAChR subunit cross-link and internalize the postsynaptic nAChR, leading to its degradation, which was shown to be the pathogenic mechanism [34,35,36,37]. Animal models of gastrointestinal hypomotility have previously been established by the intraperitoneal injection of live nAChRα3-expressing xenogeneic cells [34]. Blue dye passaging, radiochemical, and immunohistochemical evaluations demonstrated the small intestinal transit of these cells, indicating that high concentrations of nAChRα3-IgG in serum are required for intestinal nAChRα3 depletion. In addition, no loss of ganglion neurons was observed. Recently, we developed a novel murine model of autoimmune dysautonomia by nAChRα3 immunization and identified two key immunogenic peptides that could effectively prime helper T cells [38]. Physiological testing confirmed the delayed intestinal transit in these active immunized mice, and ileus occurred because of intestinal accumulation in one of the mice. It is conceivable that gnAChR antibodies act functionally on the receptors. However, further investigation is required to determine whether these antibodies have agonistic or antagonistic effects on the receptor [39]. It remains unclear how gnAChR autoantibodies are involved in gastrointestinal dysmotility in the enteric nervous system [7]. According to the results of the present study, it is clear that an important question to be resolved in the future is whether some FGIDs overlap with the AGID concept [7]. AGID is a condition that can occur at each level of the gastrointestinal tract, including the esophagus, stomach, small intestine, large intestine, rectum, or anus, and diseases at each site include achalasia, diffuse esophageal spasm, gastroparesis, pyloric stenosis, intestinal pseudo-obstruction, slow intestinal transit, colonic inertia, and anal spasm [7]. Various autoantibodies have been implicated in AGID. Moreover, in addition to gnAChR antibodies, muscle nicotinic AChR, voltage-gated potassium channel complex, voltage-gated calcium channel (P/Q type and N type), and glutamic acid decarboxylase antibodies have been previously reported [5,7,40,41]. The involvement of these autoantibodies that target the enteric nervous system in paraneoplastic neurologic syndromes including Lambert–Eaton syndrome, Chagas disease, and diabetes, leading to impaired gastrointestinal motility, has also been reported [42,43,44]. Based on previous reports, it is difficult to determine whether AGID is simply a limited form of AAG and a clinically heterogeneous group [7]. The present study indicates that some cases of FGID may be AGID. The finding that some patients with FGID have autoantibodies is important in considering the pathogenesis of FGID and, ultimately, its treatment. These antineuronal autoantibodies have been found to be present in AGIDs at each level of the gastrointestinal tract, and it is possible that some FGIDs also have antibody-positive cases. Therefore, the antineuronal autoantibodies listed here should be measured in future large studies.

This study has several limitations. It is preliminary and is an observational study with a small sample size, albeit with expert clinical diagnosis. It is necessary to confirm in the future whether gnAChR antibodies are present in a greater number of FGID patients in a prospective multicenter study. Furthermore, the LIPS assay we established was used to detect gnAChR antibodies. Recently, other new antibody assays such as flow cytometry and cell-based assays have been reported [45,46]. In addition to conventional detection methods such as radioimmunoprecipitation and LIPS assays, it is necessary to verify the presence of autoantibodies using different detection systems that incorporate other new methods. After such validation, the role of gnAChR antibodies in the pathogenesis of patients with FGID should be clarified. This will allow us to understand the true pathogenic role of this autoantibody and will provide an opportunity to investigate the possibility of immunotherapy for antibody-induced autonomic dysfunction.

In summary, we reported the presence of gnAChR antibody-positive cases of FD and IBS. These cases will be valuable in elucidating the pathophysiology of these FGIDs and developing new treatment strategies.

## Figures and Tables

**Table 1:** Clinical profiles of patients with FGID in the presence or absence of gAChR Abs.

Characteristics	FGID with gAChR Abs (n = 4)	FGID without gAChR Abs (n = 7)	*p* Value
Age (average, years)	71.5	59.7	0.262
Sex, female (%)	3 (75.0)	5 (71.3)	0.166
Mode of onset, chronic (%)	4 (100.0)	6 (85.7)	0.788
Antecedent infection (%)	0 (0.0)	1 (14.3)	0.788
Orthostatic hypotension (%)	0 (0.0)	2 (28.6)	0.527
Orthostatic intolerance (%)	1 (25.0)	4 (57.1)	0.412
Arrhythmia (%)	0 (0.0)	1 (14.3)	0.788
Pupillary abnormality (%)	0 (0.0)	0 (0.0)	1.000
Sicca (%)	3 (75.0)	0 (0.0)	0.042
Coughing episode (%)	0 (0.0)	1 (14.3)	0.788
Anhidrosis (%)	1 (25.0)	0 (0.0)	0.788
Upper GI dysfunction (%)	4 (100.0)	7 (100.0)	1.000
Lower GI dysfunction (%)	4 (100.0)	7 (100.0)	1.000
Bladder dysfunction (%)	2 (50.0)	6 (85.7)	0.412
Sexual dysfunction (%)	0 (0.0)	0 (0.0)	1.000

*p* < 0.05 was considered statistically significant. Abbreviations: FGID = functional gastrointestinal disorders; gAChR = ganglionic acetylcholine receptor; Abs = autoantibodies; GI = gastrointestinal.

**Table 2:** Comparison of the COMPASS of FGID patients with and without gAChR Abs.

Characteristics	FGID with gAChR Abs (n = 4)	FGID without gAChR Abs (n = 7)	*p* Value
COMPASS total score (average)	16.5	20.8	0.455
COMPASS orthostatic intolerance score (average)	3.0	8.7	0.294
COMPASS secretomotor score (%)	2.6	0.4	0.114
COMPASS gastrointestinal score	8.6	8.6	0.975
COMPASS bladder score	1.4	3.1	0.185

Abbreviations: COMPASS = Composite Autonomic Symptom Score; FGID = functional gastrointestinal disorders; gAChR = ganglionic acetylcholine receptor; Abs = autoantibodies.

## Data Availability

The data presented in this study are available on request from the corresponding author.

## References

[1] Koloski N.A., Talley N.J., Boyce P.M. (2002). Epidemiology and health care seeking in the functional GI disorders: A population-based study. Am. J. Gastroenterol..

[2] Black C.J., Drossman D.A., Talley N.J., Ruddy J., Ford A.C. (2020). Functional Gastrointestinal Disorders: Advances in Understanding and Management. Lancet.

[3] Vernino S., Low P.A., Fealey R.D., Stewart J.D., Farrugia G., Lennon V.A. (2000). Autoantibodies to Ganglionic Acetylcholine Receptors in Autoimmune Autonomic Neuropathies. N. Engl. J. Med..

[4] Wang Z., Low P.A., Jordan J., Freeman R., Gibbons C.H., Schroeder C., Sandroni P., Vernino S. (2007). Autoimmune autonomic ganglionopathy: IgG effects on ganglionic acetylcholine receptor current. Neurology.

[5] Dhamija R., Tan K.M., Pittock S.J., Foxx–Orenstein A., Benarroch E., Lennon V.A. (2008). Serologic profiles aiding the diagnosis of autoimmune gastrointestinal dysmotility. Clin. Gastroenterol. Hepatol..

[6] Flanagan E.P., Saito Y.A., Lennon V.A., McKeon A., Fealey R.D., Szarka L.A., Murray J.A., Foxx-Orenstein A.E., Fox J.C., Pittock S.J. (2014). Immunotherapy trial as diagnostic test in evaluating patients with presumed autoimmune gastrointestinal dysmotility. Neurogastroenterol. Motil..

[7] Nakane S., Mukaino A., Ihara E., Ogawa Y. (2021). Autoimmune gastrointestinal dysmotility: The interface between clinical immunology and neurogastroenterology. Immunol. Med..

[8] Nakane S., Mukaino A., Higuchi O., Yasuhiro M., Takamatsu K., Yamakawa M., Watari M., Tawara N., Nakahara K.-I., Kawakami A. (2020). A comprehensive analysis of the clinical characteristics and laboratory features in 179 patients with autoimmune autonomic ganglionopathy. J. Autoimmun..

[9] Nakane S., Higuchi O., Koga M., Kanda T., Murata K., Suzuki T., Kurono H., Kunimoto M., Kaida K.-I., Mukaino A. (2015). Clinical features of autoimmune autonomic ganglionopathy and the detection of subunit-specific autoantibodies to the ganglionic acetylcholine receptor in Japanese patients. PLoS ONE.

[10] Burbelo P.D., Ching K.H., Klimavicz C.M., Iadarola M.J. (2009). Antibody profiling by Luciferase Immunoprecipitation Systems (LIPS). J. Vis. Exp..

[11] Burbelo P.D., Lebovitz E.E., Notkins A.L. (2015). Luciferase immunoprecipitation systems for measuring antibodies in autoimmune and infectious diseases. Transl. Res..

[12] Maeda Y., Migita K., Higuchi O., Mukaino A., Furukawa H., Komori A., Nakamura M., Hashimoto S., Nagaoka S., Abiru S. (2016). Association between Anti-Ganglionic Nicotinic Acetylcholine Receptor (gAChR) Antibodies and HLA-DRB1 Alleles in the Japanese Population. PLoS ONE.

[13] Nakane S., Mukaino A., Higuchi O., Watari M., Maeda Y., Yamakawa M., Nakahara K., Takamatsu K., Matsuo H., Ando Y. (2018). Autoimmune autonomic ganglionopathy: An update on diagnosis and treatment. Expert Rev. Neurother..

[14] Mearin F., Lacy B.E., Chang L., Chey W.D., Lembo A.J., Simren M., Spiller R. (2016). Bowel Disorders. Gastroenterology.

[15] Drossman D.A., Hasler W.L. (2016). Rome IV—Functional GI Disorders: Disorders of Gut-Brain Interaction. Gastroenterology.

[16] Sletten D.M., Suarez G.A., Low P.A., Mandrekar J., Singer W. (2012). COMPASS 31: A Refined and Abbreviated Composite Autonomic Symptom Score. Mayo Clin. Proc..

[17] Cortez M., Reddy S.N., Goodman B., Carter J., Wingerchuk D. (2015). Autonomic symptom burden is associated with MS-related fatigue and quality of life. Mult. Scler. Relat. Disord..

[18] Mukaino A., Minami H., Isomoto H., Hamamoto H., Ihara E., Maeda Y., Higuchi O., Okanishi T., Kokudo Y., Deguchi K. (2018). Anti-ganglionic AChR antibodies in Japanese patients with motility disorders. J. Gastroenterol..

[19] Koloski N., Jones M., Walker M.M., Veysey M., Zala A., Keely S., Holtmann G., Talley N.J. (2019). Population based study: Atopy and autoimmune diseases are associated with functional dyspepsia and irritable bowel syndrome, independent of psychological distress. Aliment. Pharmacol. Ther..

[20] Svedberg P., Johansson S., Wallander M., Hamelin B., Pedersen N.L. (2002). Extra-intestinal manifestations associated with irritable bowel syndrome: A twin study. Aliment. Pharmacol. Ther..

[21] De Giorgio R., Guerrini S., Barbara G., Stanghellini V., De Ponti F., Corinaldesi R., Moses P.L., Sharkey K.A., Mawe G.M. (2004). Inflammatory neuropathies of the enteric nervous system. Gastroenterology.

[22] Kraneveld A.D., Rijnierse A., Nijkamp F.P., Garssen J. (2008). Neuro-immune interactions in inflammatory bowel disease and irritable bowel syndrome: Future therapeutic targets. Eur. J. Pharmacol..

[23] Burns G., Carroll G., Mathe A., Horvat J., Foster P., Walker M.M., Talley N.J., Keely S. (2019). Evidence for Local and Systemic Immune Activation in Functional Dyspepsia and the Irritable Bowel Syndrome: A Systematic Review. Am. J. Gastroenterol..

[24] Burns G.L., Bruce J.K., Minahan K., Mathe A., Fairlie T., Cameron R., Naudin C., Nair P.M., Potter M.D.E., Irani M.Z. (2023). Type 2 and type 17 effector cells are increased in the duodenal mucosa but not peripheral blood of patients with functional dyspepsia. Front. Immunol..

[25] De Giorgio R., Bovara M., Barbara G., Canossa M., Sarnelli G., De Ponti F., Stanghellini V., Tonini M., Cappello S., Pagnotta E. (2003). Anti-HuD-induced neuronal apoptosis underlying paraneoplastic gut dysmotility. Gastroenterology.

[26] Pittock S.J., Lennon V.A., Dege C.L., Talley N.J., Locke G.R. (2011). Neural autoantibody evaluation in functional gastrointestinal disorders: A population-based case-control study. Dig. Dis. Sci..

[27] Fan W., Fei G., Li X., Wang X., Hu C., Xin H., Sun X., Li Y., Wood J.D., Fang X. (2018). Sera with anti-enteric neuronal antibodies from patients with irritable bowel syndrome promote apoptosis in myenteric neurons of guinea pigs and human SH-Sy5Y cells. Neurogastroenterol. Motil..

[28] Fan W., Fang X., Hu C., Fei G., Xiao Q., Li Y., Li X., Wood J.D., Zhang X. (2022). Multiple rather than specific autoantibodies were identified in irritable bowel syndrome with HuProt™ proteome microarray. Front. Physiol..

[29] Bierła J.B., Cukrowska B., Skrzydło-Radomańska B., Prozorow-Król B., Kurzeja-Mirosław A., Cichoż-Lach H., Laskowska K., Sowińska A., Majsiak E. (2023). The Occurrence of Gluten-Related Antibodies, Sensitization to Selected Food Allergens, and Antibodies against Intrinsic Factor in Adult Patients with Diarrhea-Predominant Irritable Bowel Syndrome. J. Pers. Med..

[30] Volter F., Fain O., Mathieu E., Thomas M. (2004). Esophageal function and Sjögren’s syndrome. Dig. Dis. Sci..

[31] Vernino S., Lindstrom J., Hopkins S., Wang Z., Low P.A., Muscle Study Group (2008). Characterization of ganglionic acetylcholine receptor autoantibodies. J. Neuroimmunol..

[32] Wang Z., Low P.A., Vernino S. (2010). Antibody-mediated impairment and homeostatic plasticity of autonomic ganglionic synaptic transmission. Exp. Neurol..

[33] Crisp S.J., Kullmann D.M., Vincent A. (2016). Autoimmune synaptopathies. Nat. Rev. Neurosci..

[34] Meeusen J.W., Haselkorn K.E., Fryer J.P., Kryzer T.J., Gibbons S.J., Xiao Y., Lennon V.A. (2013). Gastrointestinal hypomotility with loss of enteric nicotinic acetylcholine receptors: Active immunization model in mice. Neurogastroenterol. Motil..

[35] Lennon V.A., Ermilov L.G., Szurszewski J.H., Vernino S. (2003). Immunization with neuronal nicotinic acetylcholine receptor induces neurological autoimmune disease. J. Clin. Invest..

[36] Vernino S., Low P.A., Lennon V.A. (2003). Experimental Autoimmune Autonomic Neuropathy. J. Neurophysiol..

[37] Kobayashi S., Yokoyama S., Maruta T., Negami M., Muroyama A., Mitsumoto Y., Iwasa K., Yamada M., Yoshikawa H. (2013). Autoantibody-induced internalization of nicotinic acetylcholine receptor α3 subunit exogenously expressed in human embryonic kidney cells. J. Neuroimmunol..

[38] Yamakawa M., Nakane S., Ihara E., Tawara N., Ikeda H., Igarashi Y., Komohara Y., Takamatsu K., Ikeda T., Tomita Y. (2022). A novel murine model of autoimmune dysautonomia by α3 nicotinic acetylcholine receptor immunization. Front. Neurosci..

[39] Nakane S., Yamakawa M., Nakatsuji Y. (2024). Autoantibodies against gAChR in humans and mouse models of autoimmune autonomic ganglionopathy. Clin. Exp. Neuroimmunol..

[40] Hubball A.W., Lang B., Souza M.A., Curran O.D., Martin J.E., Knowles C.H. (2012). Voltage-gated potassium channel (K(v) 1) autoantibodies in patients with chagasic gut dysmotility and distribution of K(v) 1 channels in human enteric neuromusculature (autoantibodies in GI dysmotility). Neurogastroenterol. Motil..

[41] McMillan H.J., Srinivasan J. (2010). Achalasia, chronic sensory neuropathy, and N-type calcium channel autoantibodies: Beneficial response to IVIG. Clin. J. Gastroenterol..

[42] Hirano I., Pandolfino J. (2000). Chronic intestinal pseudo-obstruction. Dig. Dis..

[43] Srinivasan S., Wiley J.W. (2000). New insights into neural injury, repair, and adaptation in visceral afferents and the enteric nervous system. Curr. Opin. Gastroenterol..

[44] Goin J.C., Sterin–Borda L., Bilder C.R., Varrica L.M., Iantorno G., Ríos M.C., Borda E. (1999). Functional implications of circulating muscarinic cholinergic receptor autoantibodies in chagasic patients with achalasia. Gastroenterology.

[45] Urriola N., Spies J.M., Blazek K., Lang B., Adelstein S. (2021). A Flow Cytometric Assay to Detect Functional Ganglionic Acetylcholine Receptor Antibodies by Immunomodulation in Autoimmune Autonomic Ganglionopathy. Front. Immunol..

[46] Karagiorgou K., Dandoulaki M., Mantegazza R., Andreetta F., Furlan R., Lindstrom J., Zisimopoulou P., Chroni E., Kokotis P., Anagnostou E. (2022). Novel Cell-Based Assay for Alpha-3 Nicotinic Receptor Antibodies Detects Antibodies Exclusively in Autoimmune Autonomic Ganglionopathy. Neurol.—Neuroimmunol. Neuroinflammation.

